# The Transcription Factor Function of Parkin: Breaking the Dogma

**DOI:** 10.3389/fnins.2018.00965

**Published:** 2019-01-15

**Authors:** Cristine Alves da Costa, Eric Duplan, Lila Rouland, Frédéric Checler

**Affiliations:** Université Côte d’Azur, Institut National de la Santé et de la Recherche Médicale, Centre National de la Recherche Scientifique, IPMC, Team Labeled “Laboratory of Excellence (LABEX) DistAlz”, Valbonne, France

**Keywords:** Parkinson’s disease, parkin, gene control, DNA binding, ubiquitin ligase, structure, transcription factor

## Abstract

*PRKN* (*PARK2*) is a key gene involved in both familial and sporadic Parkinson’s disease that encodes parkin (PK). Since its discovery by the end of the 90s, both functional and more recently, structural studies led to a consensual view of PK as an E3 ligase only. It is generally considered that this function conditions the cellular load of a subset of cytosolic proteins prone to proteasomal degradation and that a loss of E3 ligase function triggers an accumulation of potentially toxic substrates and, consequently, a neuronal loss. Furthermore, PK molecular interplay with PTEN-induced kinase 1 (PINK1), a serine threonine kinase also involved in recessive cases of Parkinson’s disease, is considered to underlie the mitophagy process. Thus, since mitochondrial homeostasis significantly governs cell health, there is a huge interest of the scientific community centered on PK function. In 2009, we have demonstrated that PK could also act as a transcription factor (TF) and induces neuroprotection *via* the downregulation of the pro-apoptotic and tumor suppressor factor, p53. Importantly, the DNA-binding properties of PK and its nuclear localization suggested an important role in the control of several genes. The duality of PK subcellular localization and of its associated ubiquitin ligase and TF functions suggests that PK could behave as a key molecular modulator of various physiological cellular signaling pathways that could be disrupted in pathological contexts. Here, we update the current knowledge on PK direct and indirect TF-mediated control of gene expression.

## Introduction

Parkin (PK) has been initially described as a ubiquitin ligase (Shimura et al., 2000), and it is clear that this function is indisputable. Biochemical, cellular, and functional clues supporting this role have been reinforced by recently delineated structural features, and notably by the identification of a RING in between RING domain that signed its membership to this class of enzyme (Riley et al., 2013; Trempe et al., 2013; Wauer and Komander, 2013). However, this molecular architecture is also the structural signature of several transcription factors (TFs). A TF is actually defined as a protein capable of binding to DNA in a sequence-specific manner and modulate gene expression levels (Fulton et al., 2009; Vaquerizas et al., 2009). Moreover, a TF should shuttle from cytosol to nuclear compartments and transit to the latter to exerts its function. PK fulfills all these criteria as discussed below. Thus, the dogmatic view of PK as a ubiquitin ligase only should likely be revisited.

## DNA-Binding Properties of Parkin

Electrophoretic mobility shift assay (EMSA) and chromatin immunoprecipitation assay (ChIP) of endogenous TF candidates are routinely considered as necessary and sufficient to delineate DNA-binding properties of a putative TF candidate. We used these two complementary approaches to demonstrate that PK downregulated *TP53* gene expression and that it physically interacted with the human promoter of *TP53* (da Costa et al., 2009; Duplan et al., 2013b). Thus, EMSA experiments using nuclear fractions of either control or PK overexpressing cells allowed us to map the PK-*hTP53* interacting domain while ChIP comparing wild-type and PK-depleted cells allowed us to validate the DNA-binding properties of endogenous PK in a natural chromatin context (da Costa et al., 2009). To definitely demonstrate that PK could directly interact with the *TP53* promoter and in order to rule out the possibilities that PK required an additional cofactor or, alternatively, acted itself as a co-factor of another TF, we have performed EMSA assay using recombinant PK and labeled synthetic DNA probes (Duplan et al., 2013b). In this bimolecular *in vitro* reaction where any protein/DNA interaction is necessarily direct, we were able to fully confirm EMSA and ChIP data and undoubtedly supported PK as a DNA-binding factor. Of most importance when data bring such type of breakthrough, our results were fully confirmed by Dr. Lipton’s team that showed that PK do repress p53 and demonstrated direct physical interaction of PK with p53 promoter by EMSA and ChIP approaches (Sunico et al., 2013).

In 2013, we have identified and validated by both approaches, two additional PK transcriptional targets linked to Alzheimer’s disease (AD), namely, presenilins 1 (PS1) and 2 (PS2) (Duplan et al., 2013b). PSs are key components of the γ-secretase complex, the enzyme involved in the production of Aβ peptides (Checler, 1995), which accumulate in senile plaques, one of the major histopathological stigmata in AD-affected brains. Here again, EMSA performed with recombinant PK showed that it bound to DNA directly without any cooperation with other TFs while ChIP assay validated the endogenous interaction in a physiological context. Interestingly, PK transactivated the PS1 promoter and repressed that of PS2 (Duplan et al., 2013b). This duality of PK TF function suggests that, as is the case for the majority of TFs, PK DNA binding to its responsive element may be impacted by surrounding sequence architecture (Rinn and Huarte, 2011). This is particularly the case for p53. Thus, it has been shown that faint sequence variations in the p53 responsive element as well as spacing between the two half-sites were important to govern p53 activity toward target activation or repression (Wang et al., 2010).

Chromatin immunoprecipitation assay on ChIP studies aimed at identifying PK targets in whole human genome led to the identification of various putative direct and indirect PK targets (Rothfuss et al., 2009). Interestingly, a substantial subset of the isolated gene sequences encoded mitochondrial proteins, suggesting an important role of PK in mitochondrial transcription/physiology in either proliferating or differentiated SH-SY5Y cells. This work was supported by a Kuroda et. al study showing that overexpressed PK interacted with mitochondrial DNA displacement loop (D-loop) sequences and stimulated mitochondrial biogenesis (Kuroda et al., 2006). Of note, interaction of PK with mitochondrial DNA *in vitro* by EMSA approach was dependent upon the presence of the mitochondrial TF TFAM (Kuroda et al., 2006), thus suggesting a TFAM-mediated indirect binding of PK to mitochondrial DNA. These EMSA assays were performed in presence of Zn^2+^ chelators like EDTA and DTT. Thus, considering that PK protein structural studies (see below) have shown an importance of zinc to its function (Trempe et al., 2013), attention should be given when performing PK *in vitro* gel shift and avoid the addition of Zn^2+^ chelators in the binding reaction to optimize PK DNA binding as illustrated for other zinc-binding proteins (Kothinti et al., 2011).

Studies aimed at testing the impact of PK on DNA repair demonstrated that PK associated with damaged DNA (Kao, 2009). This work showed that PK protected against genotoxicity *via* its interaction with proliferating cell nuclear antigen PCNA and ability to promote DNA excision repair. Importantly, ChIP experiments demonstrated that PK interacted with one of the calmodulin 1 promoter (Kao, 2009). Calmodulin 1 is the most DNA damage susceptible promoter in the human brain during aging (Lu et al., 2004). Since DNA damage is a deleterious process that accelerates during aging and a key pathological determinant in neurodegenerative disorders, one can envision an important role of PK TF function in the protection against genotoxic processes.

Overall, there are few doubts about the fact that PK could regulate gene transcription by means of its DNA-binding properties. As stated above, this could be indirect or direct. However, the above set of experimental data, and more particularly the observed direct binding of recombinant PK to p53 (Duplan et al., 2013b; Sunico et al., 2013), PS1 and PS2 synthetic probes (Duplan et al., 2013b) consistently supports the view that PK could indeed behave as a genuine TF.

## Parkin Structure: Clues for Both Ubiquitin Ligase and TF Functions

Biochemical and structural studies have identified PK as a member of the RBR (RING-in-between-RING)-containing protein family. Crystal structure determination of full length PK (Trempe et al., 2013) and its C-terminal domains (Riley et al., 2013; Trempe et al., 2013; Wauer and Komander, 2013) provided quite significant information about the role of PK domains in PK E3 ligase activity. Thus, PK structure is composed of an N-terminal ubiquitin-like domain (Ubl) and four C-terminal zinc-coordinating RING-like domains (RING0, RING1, IBR, and RING2). The Ubl domain is involved in substrate recognition, binding to SH3 and ubiquitin-interacting motif (UIM) domains, proteasome linkage, and control of cellular PK levels and activity (Finney et al., 2003; Fallon et al., 2006; Chaugule et al., 2011). The RING domains are involved in zinc ion binding through histidine and cysteine residues (Hristova et al., 2009). NMR titrations, mutagenesis, and molecular modeling identified the R1 domain as the E2-binding site (Riley et al., 2013; Wauer and Komander, 2013). The RING2 domain is the catalytic module harboring the catalytic cysteine (Cys431). The RING0 domain is exclusively found in PK structure while the IBR domain is conserved among the RBR E3 family.

The delineation of PK structure has been rightly taken as the definitive evidence that PK acted as a ubiquitin ligase. It has also sometimes led to the empirical refuting of other evidences, even when they were derived from technically sound studies. That PK could have additional functions and indeed could behave as a pleiotropic protein is not consensually accepted. This dogmatic view based on structural grounds is particularly puzzling since close examination of the literature in the field indicates that the RING finger motif can also mediate protein–DNA interactions.

First, the DNA-binding capacity of the RING1 finger domain has been specifically explored by Lovering et al. (1993). Thus, EMSA studies performed with the 55 amino acid synthetic peptide corresponding to the RING1 motif indicates its *in vitro* binding to oligonucleotides in a zinc-dependent manner since the addition of EDTA and DTT blocked the formation of specific bands. This observation raised a question. Besides its ubiquitin ligase function, could PK DNA binding be mediated by its RING1 domain? Interestingly, we firmly demonstrated that this domain was indeed involved in both p53 and PS2 transrepression and PS1 transactivation (da Costa et al., 2009; Duplan et al., 2013b).

Parkin is not the only TF whose DNA binding is mediated by a RING finger motifs. Thus, several proteins bearing this structural domain including MEL18, SNURF, LUN, RBCK1, RAD18, and MDM2 were shown to bind DNA directly by EMSA approaches (Tagawa et al., 1990; Kanno et al., 1995; Bailly et al., 1997; Tokunaga et al., 1998; Chu et al., 2001; Hakli et al., 2001; Notenboom et al., 2007; Challen et al., 2012). Interestingly three of these proteins, namely RBCK1, RAD18, and MDM2, may also function as E3 ligases. Thus, it has been shown RAD18 is implicated in the mono ubiquitinylation of a critical proliferation mediator, PCNA (Hoege et al., 2002). RBCK1 was shown to interact with ubiquitinated proteins and self-ubiquinates *in vitro* (Tatematsu et al., 2008). RBCK1 E3 ligase activity is inhibited by PKCβ phosphorylation. Interestingly, the activity of other E3 ligases are impacted by phosphorylation as has been shown for PK (Yamamoto et al., 2005). Finally, MDM2 is an E3 ligase activity and master regulator of p53 degradation by the proteasome (Haupt et al., 1997; Fang et al., 2000). Thus, it can be proposed that a group of RING motif-containing proteins could behave as both TF and ubiquitin ligase and that PK belongs to this family.

Importantly, *in silico* studies of PK protein sequence by various prediction programs (GYM, DP-bind, DNA binder, DisoRDPbind, and SMART) indicate the presence of potential RING1 independent DNA-binding motifs in the PK protein sequence, the functionality of which remains to be firmly established.

## Parkin Responsive Element

One important property of a TF stands in its ability to recognize and bind short specific sequences (6–12 bases) in the promoter region of a gene target. Usually, TFs bind to their preferred sequences referred to as consensus motif with a 1000-fold higher affinity than for other sequences (Geertz et al., 2012). Thus, the identification of a TF consensus motif is essential to the portrayal of a TF function and can be achieved by means of an arsenal of *in vitro* and *in vivo* approaches (reviewed in Jolma and Taipale, 2011). The most accurate determination of a TF consensus motif involves quantitative protein-DNA-binding affinity measurements by means of recombinant purified protein and labeled DNA probes (Stormo and Zhao, 2010). The functional validation of a consensus motif is often linked to the number of independent DNA-binding sequences (often in distinct mRNAs) in which it is found, although several TFs DNA-binding motifs were identified by means of a relatively low number (10–20) of target sequences (Mathelier et al., 2016).

Despite a considerable number of genes directly or indirectly regulated by PK (Unschuld et al., 2006; Rothfuss et al., 2009; Gonzalez-Casacuberta et al., 2018), only three (*TP53*, *PSEN1*, and *PSEN2*) have been experimentally validated by both ChIP and EMSA approaches (da Costa et al., 2009; Duplan et al., 2013b; Sunico et al., 2013). Of most interest, the examination of the DNA sequences interacting with PK allowed our team to derive a putative consensus motif corresponding to a heptamer motif (GCCGGAG). This was demonstrated by two distinct approaches. Thus, we showed that the deletion of this putative PK consensus motif from *TP53*, *PSEN1*, and *PSEN2* full-length promoter sequences coupled to the luciferase reporter gene led to a complete loss of their PK-mediated transcriptional regulation (Duplan et al., 2013b). Second, EMSA experiments using labeled p53, PS1, and PS2 DNA probes harboring or not the PK consensus motif clearly showed that the interaction of purified recombinant PK with DNA probes was drastically impacted by the ablation of the putative PK consensus motif (Duplan et al., 2013b). This network of evidences unraveled a responsive element explaining the ability of PK to control p53, PS1, and PS2 transcription. Whether this responsive element is complete remains a matter of question. Thus, although the three above-described proteins are transcriptionally regulated by PK and display strictly similar suspected responsive elements, one should keep in mind that two of them are repressed (p53 and PS2) while the other is activated (PS1). This could be interpreted in two ways. Either the consensus site is incomplete and yet unknown additional specific domains are critical to drive repression or activation processes or, alternatively, transcriptional regulation implying upstream or downstream elements contributes to the PK-mediated transcription and remains to be elucidated. The identification and validation of additional PK targets should allow refinement of the PK consensus motif. In this context, recent cutting edge high throughput technologies such as ChIP-seq (Johnson et al., 2007) will be of precious help for genome-wide identification of additional PK targets, and thus to allow comparative *in silico* studies aimed at more precisely identifying complete PK responsive element. Along with this line of reasoning, it is of most interest that close analyses of several PK transcriptional targets identified by ChIP (Kao, 2009; Rothfuss et al., 2009) indicated that they contain one or several PK consensus motifs in their promoter regions suggesting a possible direct gene regulation. As a matter of example, PK was shown to interact with the human promoter of the calmodulin 1 gene (-650/+50 from the ATG) by ChIP (Kao, 2009). Our *in silico* analysis unraveled the PK consensus motif (-206/-200 from the ATG) in the chipped sequence. This supports the view of specific PK DNA binding properties and reinforces the reliability of the PK consensus motif signature for identifying direct TF targets.

## Parkin Nuclear Localization

Subcellular studies in human and mouse brains have shown that PK is mainly localized in the cytosol in agreement with its E3 ligase function (Shimura et al., 1999), but is also observable in the nucleus (Stichel et al., 2000; Horowitz et al., 2001; da Costa et al., 2009). This dual localization fits well with the bifunctional properties of PK as ubiquitin ligase and TF. It also raises the question of how PK shuttles from one compartment to another and whether this can be affected in pathological conditions. Of most interest it has been shown that PK nitrosylation affects its localization as well as its transcriptional factor function (Sunico et al., 2013). Thus, PK nitrosylation leads to its sequestration in the cytosol and by consequence the impairment of its physical interaction with the *TP53* promoter (Sunico et al., 2013). The lack of transcriptional repression of the pro-apoptotic protein p53 by PK could well contribute to the exacerbated neuronal loss observed in PD (Checler and Alves da Costa, 2014). Since nitrosylation processes mediated by environmental stressors are increased in sporadic PD (Gu et al., 2005; Vance et al., 2010), the blockade of PK TF function by nitrosylation suggests that PK-mediated gene control may well contribute to the physiopathology of sporadic PD.

Other post-translational events could impact PK subcellular localization. SUMOylation has been proposed as a cellular means to control subcellular localization of target proteins (Mahajan et al., 1997; Muller et al., 1998; Duprez et al., 1999). PK protein interaction with the RanBP2 SUMO E3 ligase (Um et al., 2006) suggested that it would be SUMOylated and indeed, PK was shown to associate with SUMO-1. Of most interest, PK SUMOylation was shown to impact its localization by favoring its shuttling to the nucleus (Um and Chung, 2006). The fact that SUMOylation processes have been associated with neuroprotective phenotypes in PD (Vijayakumaran and Pountney, 2018) suggests that PK transcriptional nuclear function contributes to PK-induced neuroprotection, a conclusion that agrees well with the PK-mediated transcriptional repression of the cell death inducer p53. The deleterious impact of impaired basal SUMOylation has been observed in various neurodegenerative diseases including PD (Cho et al., 2015; Matsuzaki et al., 2015). Thus, it would be quite interesting to investigate if PK SUMOylation is decreased in sporadic PD.

It has also been shown that DNA damage triggers PK translocation to the nucleus (Kao, 2009). This should allow PK-mediated transcriptional control of genes involved in DNA repair, notably calmodulin 1. As stated above, calmodulin1 is a one of the most impacted human genes affected by DNA damage during aging (Lu et al., 2004). Since aging is a major risk factor for PD (Reeve et al., 2014), one can envision that PK TF function may underlie an important defense cellular mechanism against genotoxicity.

Finally, it has been shown that PK missense mutations may also alter its localization (Cookson et al., 2003). Thus, the R256C and R275W PK mutants were observed in cytosolic and nuclear inclusions. Interestingly, PK nuclear inclusions were particularly associated with the R256C mutation and PK expression did not merge with YOYO-1 DNA staining, suggesting an impact of PK mutations on PK-mediated DNA binding. Thus, the compartmentalization/sequestration of PK in nuclear inclusions probably impedes its access to DNA. Moreover, we have shown that other familial PK mutations (K161N and C441R) impact PK transcriptional function by interfering with the PK DNA binding properties. Thus, purified recombinant PK mutants do not interact with *TP53*, *PS1*, and *PS2* promoter-derived sequences in EMSA assay (Duplan et al., 2013b).

## Classification of PK Gene Targets and Associated Biological Functions

**FIGURE 1:**
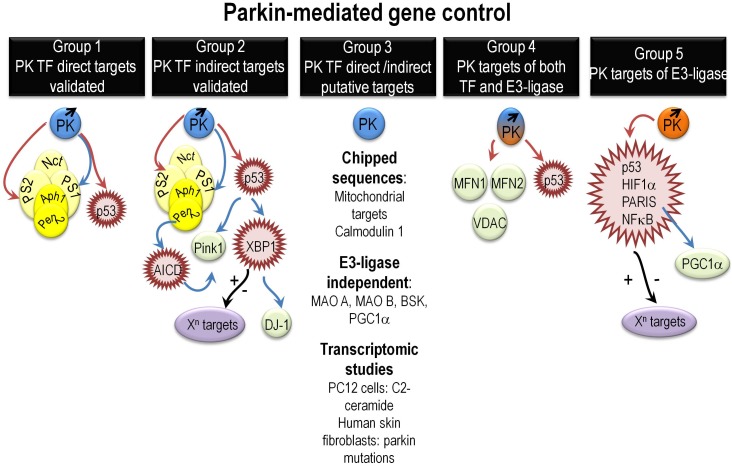
Overview of various mechanisms governing PK-mediated direct or indirect gene regulation. It summarizes the various mechanisms underlying direct or indirect PK-mediated gene regulation as extensively described in the “PK gene targets and associated biological functions” paragraph. Abbreviations that were not already described in the main text include Aph1 (anterior pharynx defective), Pen2 (presenilin enhancer-2), and AICD (Amyloid precursor protein intracellular domain). Red, blue, and black arrows indicate repression, activation, and yet unknown gene control phenotypes, respectively.

Overall, close inspection of the literature allowed us to distinguish five groups of PK-mediated regulated genes (Figure 1).

Group 1 gathers targets regulated by direct PK-mediated DNA binding to their promoter sequences and validated by both ChIP and EMSA approaches (da Costa et al., 2009; Duplan et al., 2013b; Sunico et al., 2013). They are essentially p53, PS1, and PS2. This reinforces in the view that PK could well contribute to functions altered in PD but also in other neurodegenerative diseases and cancer. Thus, PSs are at the center of gravity of AD (Checler, 1999; De Strooper et al., 2012; Duggan and McCarthy, 2016) and p53 is a key pro-apoptotic mediator, the activity of which is exacerbated in neurodegenerative diseases and extinguished by loss of function mutations in an important subset of cancers (Checler and Alves da Costa, 2014).

Group 2 brings together indirect PK transcriptional targets that could themselves behave or not as TF. We have shown that PK upregulates PINK1 transcription indirectly *via* PS1 and p53 (Goiran et al., 2018a,b). PINK1 is a key PD causative gene that controls the mitophagy process dependently or independently of PK (Youle and Narendra, 2011; de Vries and Przedborski, 2012). The regulation of PINK1 by PK *via* the control of PS reinforces its role in AD and allows a better understanding of the mitophagic failure in AD. Taken as a whole, the above-cited works indicate that there is a feedback loop regulation between PK and PINK1 and by consequence that PK can also act upstream of PINK1 (Goiran et al., 2018a).

Parkin transcriptionally upregulates another PD causative gene product DJ-1(Duplan et al., 2013a). This regulation is driven by PK transcriptional repression of *TP53*. PK *TP53* repression leads to the upregulation of another master TF involved in unfolded protein response (UPR), XBP1 (X box-binding protein). XBP1 directly binds to DJ-1 promoter and regulates DJ-1 transcription (Duplan et al., 2013a). The regulation of XBP1 by PK highlights its contribution the UPR control and reinforces the importance of the UPR in PD pathophysiology. Moreover, since XBP1 transcriptional function is involved in a wide range of biological processes, one can speculate on the upstream influence of PK in these various XBP1-dependent phenotypes.

Group 3 assembles potential direct (DNA binding) PK-regulated genes awaiting definitive demonstration. These targets include all PK targets identified by ChIP on ChIP approaches (Rothfuss et al., 2009). Of note, the authors highlighted that most of PK targets were of mitochondrial origin suggesting an important role of nuclear PK in mitochondrial homeostasis. This group also includes PK-regulated genes encoding protein, the degradation of which does not involve PK E3 ligase activity like monoamino oxidases A and B (Jiang et al., 2006), PGC-1α (Zheng et al., 2017), and the c-Jun N-terminal kinase (JNK) drosophila homologue gene BSK (basket) (Hwang et al., 2010). Finally, this large group gathers potential PK targets identified by microarray. Thus, transcriptomic studies performed in PC12 cells demonstrated that numerous genes linked to apoptosis and cellular stress were induced by C2-ceramide and that PK overexpression triggered cytoprotection *via* the reduction of EIF4BP1, GADD45A, and PTPN-5 mRNA levels (Unschuld et al., 2006). A RNAseq study of skin fibroblasts from either healthy or PD patients carrying PK mutations show a modulation of 343 genes, which function and gene ontology indicate a link of PK with cell adhesion, cell growth, and amino acid and folate metabolism among others (Gonzalez-Casacuberta et al., 2018).

Group 4 highlights the possible dual regulation of some genes and their translation products by both PK TF and E3 ligase activities, respectively. Thus, in this group, one can find p53, mitofusin 1, mitofusin 2, and voltage-dependent anion channel 1 (VDAC) that are validated E3 ligase substrates and potential PK TF targets (Geisler et al., 2010; Glauser et al., 2011; Chen and Dorn, 2013).

Group 5 gathers PK E3 ligase substrates that harbor a TF function like p53, NFkB, HIF1α, and PARIS (ZNF746) (Henn et al., 2007; Shin et al., 2011; Jung et al., 2017; Liu et al., 2017). Given the enormous network of genes regulated by these TFs, one can envision a huge impact of PK in a variety of cellular functions. It should be noted that *TP53* is also a TF target of PK suggesting that both TF and ubiquitin ligase functions may be implicated in transcriptional and post-traductional control of a given target. Conversely, some of the above-cited proteins could well behave as PK E3 ligase substrates only, without being transcriptionally regulated by PK.

## Concluding Remarks and Future Directions

It is clear that PK harbors ubiquitin ligase and TF functions, which could be both involved in gene regulation. Delineating the respective contribution of these functions in physiological and pathological conditions should help to understand the cellular defects underlying both neurodegenerative and cancer pathologies and should allow the development of innovative diagnostic and therapeutic tools centered on its direct and/or indirect gene control.

## Author Contributions

All authors listed have made a substantial, direct, and intellectual contribution to the work and approved it for publication.

## Conflict of Interest Statement

The authors declare that the research was conducted in the absence of any commercial or financial relationships that could be construed as a potential conflict of interest.
